# Investigation of *Toxoplasma gondii* in wastewater and surface water in the Qinghai-Tibet Plateau, China using real-time PCR and multilocus genotyping

**DOI:** 10.1038/s41598-022-09166-0

**Published:** 2022-03-31

**Authors:** Anna Lass, Ioannis Kontogeorgos, Liqing Ma, Xueyong Zhang, Xiuping Li, Panagiotis Karanis

**Affiliations:** 1 Department of Tropical Parasitology, Institute of Maritime and Tropical Medicine in Gdynia, Medical University of Gdansk, 9b Powstania Styczniowego Str., 81-519 Gdynia, Poland; 2 State Key Laboratory of Plateau Ecology and Agriculture, Center for Biomedicine and Infectious Disease, Qinghai University, 1#Wei’er Road, Qinghai Biological Scientific Estate Garden, Xining, 810016 People’s Republic of China; 3 Marine Sciences Department, School of Environment, University of the Aegean, University Hill, 88 100 Mytilene, Greece; 4 Medical Faculty and University Hospital, Cologne University, Cologne, Germany; 5 Medical School, Department of Basic and Clinical Science, University of Nicosia, 93 Agiou Nikolaou, 2408 Nicosia, Cyprus

**Keywords:** Risk factors, Environmental microbiology, Parasitology

## Abstract

*Toxoplasma gondii* is a protozoan parasite, causing one of the most prevalent parasitic infections in the world. In the present study water sources of the Qinghai-Tibet Plateau (QTP), China, where the hygienic infrastructure is still developing, were investigated. A total of 214 water samples of 10 L volume, were collected from wastewater treatment plants (WWTPs), a slaughterhouse and rivers. The samples were filtered and then analysed using real-time PCR and multilocus genotyping. *T. gondii* DNA was found in four (1.9%) samples representing *T. gondii* type I; in one of them *T. gondii*-like oocysts were also confirmed microscopically. The approximate level of contamination of positive samples ranged between 30 and 2300 T*. gondii* sporozoites. The results of this study confirmed that *T. gondii* is present in wastewater in the greater metropolitan area of Xining and a neighbouring county. Contamination of wastewater at this level constitutes rather a moderate source of *Toxoplasma* infections in humans and animals. It suggests, however, a link between environmental exposure of animals, meat processing facilities and WWTPs. To our knowledge, this is the first investigation describing *T. gondii* detection in wastewater and environmental water samples collected from the territory of P.R. China using sensitive molecular tools.

## Introduction

*Toxoplasma gondii* is a worldwide distributed protozoan parasite that can infect humans as well as all warm-blooded animals. In the majority of cases, toxoplasmosis is asymptomatic in immunocompetent individuals; it may have, however, significant repercussions in immunodeficient patients and immature foetuses or infants whose mother suffered from primary infection during pregnancy^1^. Toxoplasmosis is one of the most prevalent parasitic infections in humans. Various authors suggest that one-tenth up to half the human population has been chronically infected with *T. gondii* in a global scale^2,3^. Humans acquire the disease mainly via the oral route through consumption of raw or undercooked meat of infected animals containing cysts filled with parasites^4^ and through the ingestion of oocysts as a result of drinking contaminated water, eating contaminated food or contact with contaminated soil^3,5^. Thus, the contaminated environment may be an important source of human exposure. Oocysts are excreted to the environment only by infected felids, being the definitive hosts of *T. gondii*, therefore, cats play a significant role in the epidemiology of toxoplasmosis^6,7^.

Waterborne toxoplasmosis presents a considerable threat to public health throughout the world^8,9^. *T. gondii* oocysts has been detected in water samples, in different parts of the world, including France^10^, Poland^11^, Germany^12^, Russia, Bulgaria^13^, Scotland^14^, Turkey^15^, Brazil^16^, Ecuador^17^. There is limited number of studies, however, reporting the presence of *T. gondii* in wastewater. DNA of the parasite has been detected in Bulgaria in raw sewage^13^ and in Germany in both the influent and effluent of a WWTP^12^; in other studies performed in Greece, Bulgaria, France and Germany^18–20^ *Toxoplasma* has been examined but not detected. Regarding quantitative analysis, even fever studies have been published for *Toxoplasma* in water, including the study conducted in Brazil^21^.

In China, scientific interest in *T. gondii* is visible, regarding, however, epidemiological studies of *T. gondii* research has primarily revolved around high risk populations and animals. The most recent status of toxoplasmosis in China was revealed with the nationwide survey for parasites in human population conducted between 2001 and 2004. The survey resulted in an average national prevalence rate of *T. gondii* infection of 7.9% out of a 47,444 population sample^22^ indicated that in China the national infection rate is well below the world average. Also many studies have been conducted regarding the prevalence of *T. gondii* in commercially raised livestock^23–28^ and in meat tissue sampled from meat available in retail stores, particularly pork^29^ and poultry^30^; as well as cats and dogs both companion and stray^31–33^. Even though *T. gondii* is being investigated in China, only a few studies have examined the presence of this parasite in the environment, mostly in soil^34–38^. Regarding studies performed in the Qinghai Tibetan Plateau (QTP), 12.7% of 268 soil samples taken from urban agglomerations in Qinghai and Gansu provinces, including the two respective capitals, Xining and Lanzhou were found positive for *T. gondii*^39^. In addition, a recent study carried out in QTP revealed that fresh vegetables offered at open markets in Qinghai province may be contaminated with *T. gondii*^40^. Thus, above studies demonstrate that *T. gondii* is present in the environment in different parts of China. However, to our knowledge, there are no environmental studies related to the presence of *T. gondii* in waters in China and therefore possible contribution of this medium in transmission of the parasite is unknown.

Globally, the clonal population structure of *T. gondii* encompass three major types I, II and III frequently observed in North America and Europe^41^. Atypical types were observed in wildlife in North America^42^ as well as in humans and animals in South America^43,44^. In China, type China 1 (ToxoDB#9) is widespread and likely the major lineage mainly in Northern, Southern and Central parts of the country^45–48^. The second most commonly found genotype in China is the type I (ToxoDB#10) that predominates in the eastern and southern provinces of China. It has also been found in the north-western China, including Qinghai Province^45,49^. *Toxoplasma* genotypes belonging to type II are less commonly detected in China, mostly in the northern, north-western and central provinces^45^.

The present study aimed to determine the possible occurrence of *T. gondii* in water and wastewater samples collected from Qinghai Province, P.R. China. Sensitive molecular tools (real-time PCR, qPCR, multilocus PCR/RFPL analysis) were used to determine the genotype of obtained *T. gondii* isolates and the equivalent of a number of parasites in investigated samples.

## Results

**Figure 1:**
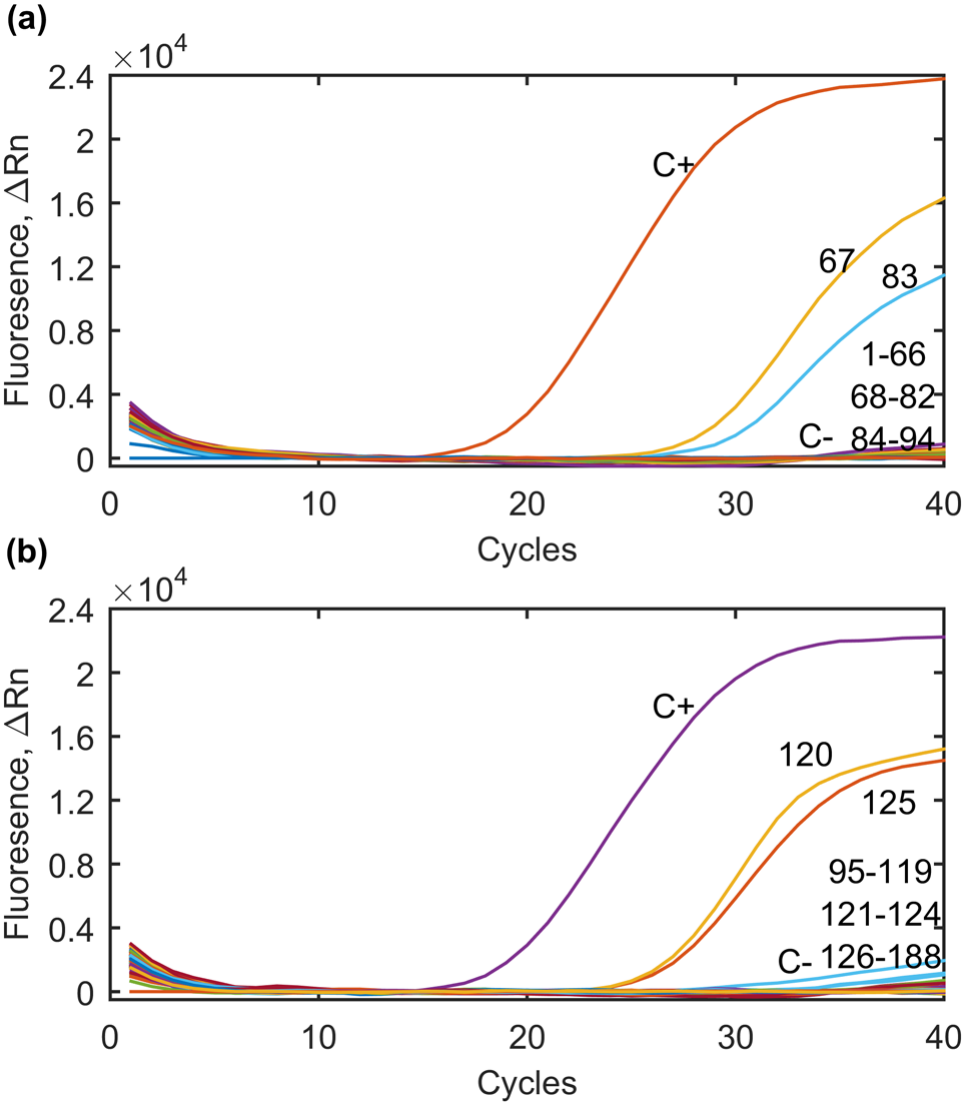
Results of the *Toxoplasma gondii* DNA detection in water samples collected from the area of Qinghai Province, P.R. China using real-time PCR. A) Results of real-time PCR performed for samples 1-94; C+-*T. gondii* positive control, 67-positive wastewater sample (template No. 67), 83-positive wastewater sample (template No. 83), C--negative control. B) Results of real-time PCR performed for samples 95-188; C+-*T. gondii* positive control, 120-positive wastewater sample (template No. 120), 125-positive wastewater sample (template No. 125), C--negative control. Figure was prepared using MATLAB R2015a.

In total, 214 water and wastewater samples were collected and examined with real-time PCR assays based on the *T. gondii* B1 gene. The presence of *T. gondii* DNA was recorded in four samples investigated (4/214; 1.9%), including three samples collected from WWTPs and one sample collected from the slaughterhouse (Table 1, Fig. 1). In case of one positive sample collected from WWTPs, presence of *T. gondii*-like oocysts was also confirmed microscopically; oocysts appear as cystic structures, approximately 10 μm in diameter (Table 1, Supplementary Fig. S1). Sequencing of positive water samples showed that the PCR products were fragments of the *T. gondii* B1 gene. *T. gondii* DNA was not found in water samples collected from surface waters (Table 1, Fig. 1). Positive samples were collected in April and August (Table 2); samples collected during the months of January, March, May, September and October were negative.

**Table 1:** Summarized results on the detection of *Toxoplasma gondii* DNA in water and wastewater samples collected from different sources in Qinghai Province, P.R. China using real-time PCR.

Sampling site	No. of samples investigated	No. of positive samples (%)	95% CI
Wastewater	188	4 (2.1)	0.8–5.3
Xining WWTP	38	1 (2.6)	0.5–13.5
Huangyuan WWTP	101	2 (2)	0.6–7.0
Baide slaughterhouse	49	1 (2)	0.3–10.6
Surface water	26	0	
Guoluo River	10	0	
Chahan River	9	0	
Baoku River	7	0	
Total	214	4 (1.9)	0.7–4.8

**Table 2:** Characteristics of the positive water samples collected from different water sources in Qinghai Province, P.R. China, regarding sampling time, reproducibility of real-time PCR results and the determined *Toxoplasma* genotype.

Template No	Sampling site	Sampling month	Microscopy	Reproducibility of real-time PCR	qPCR results						Type
					Cq (ΔR) av	Cq (ΔR) SD	NP	OCS	SCS	CCS	
67	Xining WWTP	April	–	3/3	30.3	0.1	1.19e + 002	4	8	34	I
83	Baide slaughterhouse	April	–	3/3	27.7	0.06	7.00e + 002	25	50	200	I
120	Huangyuan WWTP	August	–	3/3	26.3	0.13	1.84e + 003	65	130	520	I
125	Huangyuan WWTP	August	–	3/3	24.1	0.03	8.27e + 003	295	590	2360	ND

(3/3) three positive results obtained per three repeats of real-time PCR.

*ND* not determined, *Cq (ΔR) av*. average Cq values obtained from triplicates of positive water samples, *Cq (ΔR) SD* values of standard deviation obtained from triplicates of positive water samples, *NP* initial copy number (number of copies of *T. gondii* B1 gene) present in positive water samples calculated based on the standard curve, *OCS* the equivalent of the initial number of *T. gondii* oocysts present in positive water samples. SCS – the equivalent of the initial number of *T. gondii* sporocysts present in positive water samples (OCS × 2, one oocyst consist of two sporocysts). *CCS* equivalent of initial number of *T. gondii* cells (sporozoites) present in positive water samples (OCS × 8, one oocyst consist of two sporocysts filled with four sporozoites each).

Among the wastewater samples tested, four were positive for *T. gondii* (4/188; 2.1%) (Table 1). Parasite DNA was detected in one sample collected from the Xining WWTP (1/38; 2.6%), and in two other samples collected from the Huangyuan WWTP (2/101; 2%). Among the samples collected from the slaughterhouse, one was positive for *T. gondii* (1/49; 2%). None of the 26 samples collected from rivers located in the Qinghai Province, were positive for *T. gondii* (Table 1).

**Figure 2:**
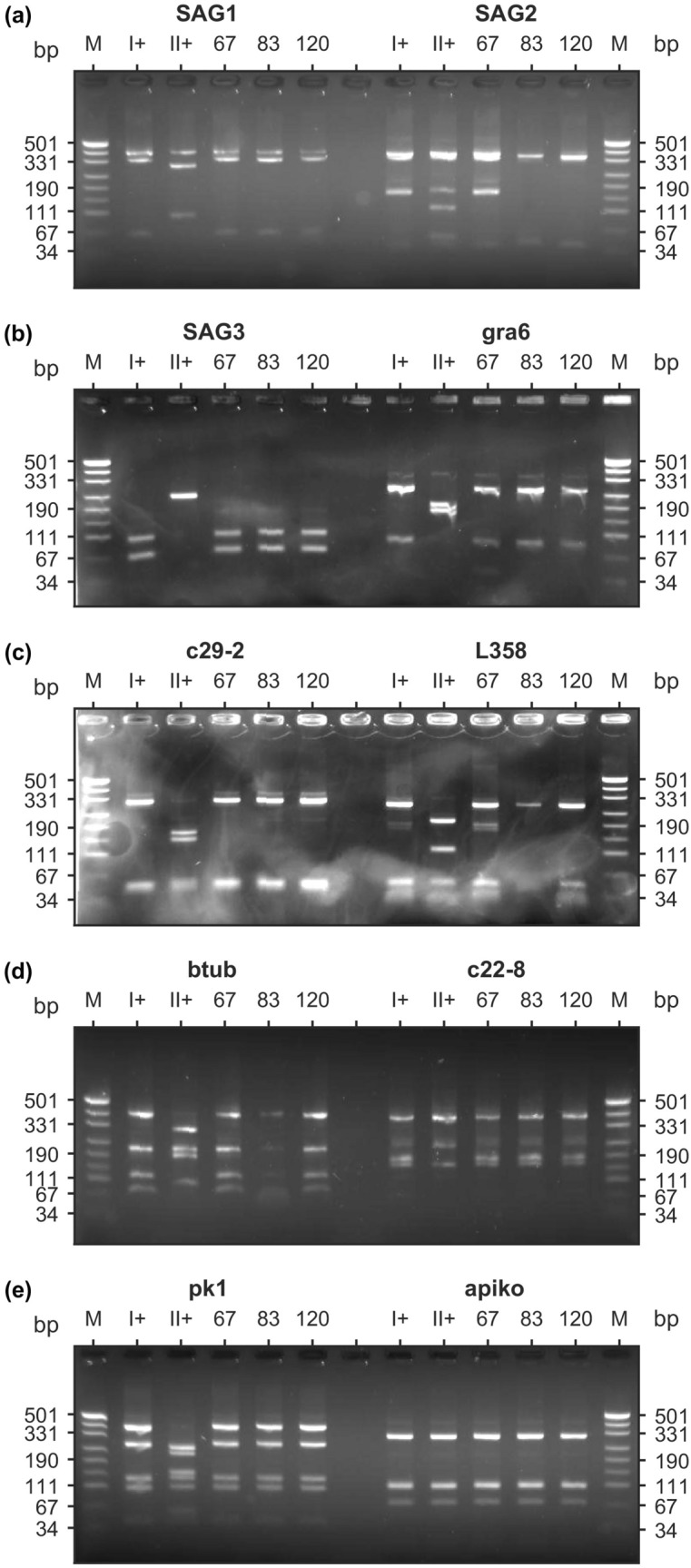
Results of multiplex multilocus nested PCR-RFLP (Mn-PCR-RFLP) analysis of Toxoplasma gondii samples using 10 different genetic markers. M (pUC19 DNA/MspI (HpaII) Marker, Thermo Fisher Scientific) – molecular weight marker, I+ – *T. gondii* type I positive control, II+ – *T. gondii* type II positive control, 67 – positive wastewater sample (template No. 67), 83 – positive wastewater sample (template No. 83), 120 – positive wastewater sample (template No. 120). The samples derive from the same experiment and gels were processed in parallel. The gels were properly cropped to improve the clarity and conciseness of the presentation; the full-length gels are presented in Supplementary Fig. S2–S5. Figure was prepared using MATLAB R2015a.

It was possible to perform full multilocus genotyping of three out of four positive wastewater samples. PCR/RFLP analysis based on a set of 10 genetic markers showed that all detected isolates represent *T. gondii* genotype I (Table 2, Fig. 2). The pattern obtained presented in Fig. 2 is clearly different from other strains found in China^50–52^.

Results of quantitative real-time PCR enabled the calculation of equivalent of *T. gondii* cells and oocysts present in positive wastewater samples, in the final suspension obtained after concentration procedure. The approximate level of contamination of positive samples ranged between 32 and 2360 *Toxoplasma* cells which correspond with respectively 4 and 295 T*. gondii* oocysts per sample (Table 2).

## Discussion

In the present study, we successfully detected *Toxoplasma* DNA in wastewater samples from QTP in China. To the best of our knowledge, this is the first investigation describing *T. gondii* detection in environmental water samples collected from the territory of P.R. China using sensitive molecular tools, supported by microscopy. *T. gondii* DNA was detected in four wastewater samples (4/188; 2.1%), originating from WWTPs inlets, before treatment, and a slaughterhouse located in the vicinity of Xining, Qinghai Province, P.R. China. In one of these positive samples we confirmed presence of *T. gondii*-like oocysts by microscopic investigation. The results of the study confirmed that *T. gondii* was present in the wastewater of two cities in QTP. This is an indicator of possible contamination of water sources in QTP since *Toxoplasma*^12^ and other protozoan parasites^53–58^ have been found in the effluent of WWTPs. Previous studies performed specifically in the area of QTP have revealed the presence of different protozoan parasites including *Acanthamoeba*, *Giardia*, *Cryptosporidium* in both surface waters and wastewaters^57,59,60^. In Xining City since 2000, a major effort has been put in the modernization of the wastewater treatment facilities, including the construction of new plants and the modernization of older ones^61^; this is indicating lack of wastewater treatment facilities the previous decades as well as lack of such facilities in smaller cities and communities in the countryside. Simultaneously, almost half the water supply for the city of Xining comes from surface water sources^62^, therefore, the presence of *T. gondii* in wastewater constitutes a potential risk for toxoplasmosis in humans and animals in the investigated area. The level of positive wastewater samples obtained in this study, however, indicates that this risk may be relatively low. It can also be suggested that water contaminated with oocysts may serve as indirect source of infection in humans when used for irrigation, washing and processing of agricultural products. Many studies performed in different parts of the world have already confirmed presence of *Toxoplasma* on agricultural products indicating irrigation as one of possible sources of contamination^63–65^. A concurrent with this study investigation, conducted in Xining open markets particularly, presented a contamination level of 3.6% of fresh vegetables samples originating from different parts of China^40^. The irrigational water is considered as a plausible contamination pathway for such vegetables and in the case of surface water used as a source, loadings from WWTPs further exacerbate the contamination.

The overall rate of positive for *T. gondii* samples collected from WWTPs in QTP, China was 2.2% (3/139). Presence of *Toxoplasma* in wastewaters was previously confirmed in Bulgaria^13^ and in Germany^12^. In other studies performed in Paris, Hamburg, Bulgaria and Greece, wastewater was tested but *T. gondii* was not detected^18–20^. All of these studies, however, have differences in the sampling, concentration and identification methods affecting their detection sensitivity and their results are subject to the specific epidemiological conditions. Therefore level of contamination established in our study cannot be simply compared to results obtained in cited articles. Until now, there is no established one sensitive protocol for detection of *T. gondii* oocysts in water. We can presume that oocysts, if present, are dispersed in waters in low numbers. Therefore, to fulfil basic purpose of our study, which was to check possibility of presence of *T. gondii* in water and wastewater, it was legitimated to select more sensitive and specific method, which is PCR. Microscopy, characterized by lower detection limit, has been added only to complement molecular techniques.

Both WWTPs in this study perform secondary treatment but differ on the connected sewer system; the Huangyuan WWTP treats wastewater from a combined sewer system, while the Xining WWTP is connected to a separate sewer system. This specification renders the Huangyuan WWTP more vulnerable to *T. gondii* contamination since in an event of overload of the capacity of the plant, e.g. during extreme rainfall events, the WWTP is bypassed and wastewater is disposed of in the river untreated. Furthermore, presence of *T. gondii* in this WWTP may be a result of environmental pollution of the streets and open spaces of the city of Huangyuan, which the WWTP is accommodating, owning to the combined sewer system. Surface runoff may be transporting *T. gondii* oocysts in the sewer system. In the study performed in Xining City and other cities of QTP, *T. gondii* presence was reported in soil samples taken from green spaces^39^. There were no positive samples in Huangyuan County, possibly due to the examination of only 10 samples from the particular district, however, whenever an adequate number of samples was taken (> 30), prevalence rate was between 11 and 16%, including the city of Lanzhou that is situated in the borders of QTP^39^. In a study from 2012 it has been suggested that felids such as Pallas’s cat (*Otocolobus manul*) and Chinese mountain cat (*Felis bieti*) may be sources of *T. gondii* oocysts in the environment of the QTP area^66^. Although there is no study for cats in Qinghai Province, in Lanzhou City, Gansu Province, which is a major city in the same region and very close to Xining, several investigations reported the presence of *T. gondii* in cats^31,67–69^. Taking into account the presence of cats in an urban environment and their role as definitive hosts of *T. gondii*, contamination of the urban environment by cats may be considered as a putative source of *Toxoplasma* in the sewer system of Huangyuan.

Similarly, contamination of wastewater with *Toxoplasma* in Xining City may be a result of contaminated wastewater coming from slaughterhouses like the one examined in this study, as it has been recently confirmed for *Cryptosporidium*^57^. Infected animals, meat and meat processing by-products are the primary sources; during the washing process, water transports *T. gondii* into the sewer system, the WWTP and possibly the receiving waterbody, Huangshui River. Some studies have reported *T. gondii* prevalence in the two most commonly bred animals in QTP, intended for human consumption, sheep^27^ and yak^66,70^. These animals free-graze in the high pasturelands, living closely together with other domesticated and wild animals, including felids and have therefore chances to ingest *T. gondii* oocysts through the faecal-oral route. Therefore, infected meat could be an important source of infection for humans in QTP and other parts of China supplied with meat from QTP. Apart from infected meet, another source of *Toxoplasma* in the wastewater samples of the slaughterhouse could have been contamination of processing area with oocysts of the parasite. We can speculate with great caution that free-grazing animals may transport contaminated soil and/or cats faeces from the pasture area on their body surface, their hooves or legs for example. However, taking into account very limited data regarding presence of *T. gondii* oocysts on the body of animals in general^71^, to verify this hypothesis additional wider studies should be carried out on this type of transportation in livestock. Moreover, we cannot exclude presence of stray cats eating unwanted tissues from positive animals and subsequently contaminating the area of slaughterhouse with oocysts. Contamination of the wastewater sample originating from the slaughterhouse is not surprising and suggests further dispersion of contamination into the wastewater treatment system and possibly water supplies.

All water samples taken from rivers were negative. According to literature, the range of positive *T. gondii* surface water samples ranges from 0 to 9% in studies conducted in developed countries, and up to 77% in low and middle income countries^72^. This fact has to be addressed with caution because the use of different methods for sampling, concentration, detection of oocysts together with epidemiological conditions makes direct comparison difficult. Most likely *T. gondii* oocysts, if present, are widely dispersed in running water and in such low numbers that it was not possible to obtain any positive result due to the sampling method limitations in conjunction with detection limits of the applied concentration method. Sampling was done in 10 L batches; this corresponds to low sampling volume and a very narrow timeframe of detection. Recovery losses during concentration method enhance the limitations of the methodology and detection strategy. In retrospect, a continuous sampling method may have produced different results. This method can provide an adequate number of samples to conclude on the prevalence of *Toxoplasma* oocysts in rivers, although such a method presents technical challenges that are difficult to overcome, especially in remote areas. It should be also highlighted that in this particular study we investigated a relatively low number of surface water samples which may not fully reflect reality. Therefore, additional studies screening larger volume surface water are needed in the future to draw unequivocal conclusions about the presence of *T. gondii* in water supplies in this area.

An approximation of the *Toxoplasma* charge was performed using qPCR. The order of magnitude of the number of oocysts that might be present in the positive samples ranged from one to 10^2^. Actual contamination, however, could be even higher than demonstrated due to possible loss of material during the purification and recovery process. The number of oocysts responsible for the development of infection in humans is unknown and the examination of the viability of oocysts was out of the scope of this study, therefore we cannot comment on the infectiousness of the oocysts in water samples. Quantitative analysis, that helps to estimate approximate oocysts load of the samples, however, can be combined with infectiousness in the future and should not be neglected, even though currently only a few studies^21^ have performed such analyses regarding *Toxoplasma* in water.

Multilocus genotyping revealed that the detected isolates in this study represent the *T. gondii* genotype I (Fig. 2). According to Chaichan et al. (2017) in China, thirteen ToxoDB genotypes and 5 atypical genotypes were identified so far^45^. Among them, genotype Chinese 1 predominates^45,47,73^, displaying a decreasing gradient from east to west^45^. When comparing gel electrophoresis images, type I^74,75^ is distinctively different from the type Chinese 1^51,52^ which is more similar to type II^52^. It is the second most common genotype found in China characterising around 15% of the isolates^45,73^. Interestingly, type I of *T. gondii* has been reported in the area of QTP (Northwest of China) including Gansu and Qinghai Province in wildlife, Plateau pika (*Ochotona curzoniae*) and Qinghai vole (*Microtus fuscus*)^45,49^. The pattern of the genotype distribution in QTP differs from the rest of China and is most probably connected with the subject of the studies conducted so far. These include domestic birds that show a predominance of type II genotype^50^ while they don’t include domestic animals and cats where Chinese 1 is expected to be more prevalent in China^25,45–47,51^. One such study in Gansu regarding free ranging white yaks (*Bos grunniens*) with limited results, has resulted in two cases of Chinese 1^76^. The aforementioned argument is further substantiated by the detection of type Chinese 1 in soil samples from Gansu^77^. On the contrary, Zhang et al. (2013) have suggested that presence of *Toxoplasma* type I in animals living in QTP area may depend on the unique environment in this high altitude plateau^49^. In any case, type I from the wildlife reservoir, somehow has to be transported to or transmitted within the urban environment since it is now reported in wastewater. Therefore more research needs to be done towards the genetic characterization of *T. gondii* in this high-altitude environment including both different hosts as well as environmental matrices; this study further adds some evidence to support the prevalence of *Toxoplasma* type I in QTP.

The waterborne transmission route of *T. gondii* to humans via the dissemination of oocysts through surface water and its epidemiological impact has been shown to be more significant than previously believed^8^. Several waterborne outbreaks have been reported worldwide since 1979, especially in Brazil, where contaminated drinking water was vehicle of infection in humans^63,64^. Oocysts are very resistant to unfavourable environmental conditions and to chemical deactivation, which makes treatment process difficult^7^. In the present paper, we describe the detection of *T. gondii* DNA in wastewater samples in QTP, China, and identified the *Toxoplasma* genotype, that is known to be pathogenic for humans. Therefore, we provide evidence for the presence of *Toxoplasma* in wastewater indicating not only the presence of this parasite in the environment in QTP but also the potential risk for humans and animals. In order to estimate, however, the infectivity of putative oocysts in the samples and their ability to infect humans and/or animals, detection of DNA itself is not enough and must be complemented by additional infectivity tests. The amount of viable and infective *Toxoplasma* oocysts in this environment remains unknown. It is also noted that in the Xining metropolitan area a wastewater reuse program has been programmed, including irrigation and various other purposes, like street cleaning^61,62^. In order to ascertain the extent of the risk for humans, future monitoring should include widespread and regular sampling from both the inlet and the outlet of WWTPs, surface waters, particularly lakes and pools where the parasite is more likely to be detected, water treatment facilities, the water supply network and could be expanded to other environmental matrices, such as soil. Moreover, finding of *T. gondii* in the washing water from a slaughterhouse calls for stricter control and monitoring of the meat-producing industry regarding protozoan parasites. It is also advised that food crops already susceptible to *T. gondii* contamination like fruits and vegetables^78^, have to be regularly and well inspected, especially in the cases of reclaimed wastewater used as irrigational water. With the new global goals announcement by the Chinese government for sustainable development in the country, it is high time for a concerted effort to tackle the prevalence of toxoplasmosis and other neglected diseases caused by food- and waterborne pathogens.

## Conclusions

Toxoplasmosis remains a disconcerting problem for public health in China as well as in other parts of the world. Current methodologies for detection of *Toxoplasma* in environmental waters, regardless of their limitations, are readily available for application. In the present study, we reported wastewater contamination with *T. gondii* in Qinghai-Tibet Plateau using sensitive molecular tools. These findings, together with previous studies showing contamination of the soil and vegetables are an indicator of possible contamination of water sources in QTP with *Toxoplasma*. On the other hand, the level of contamination of investigated wastewater samples in our study is relatively low in comparison with other countries. This corresponds with the generally low level of *Toxoplasma* infection within Chinese society as compared with the world average. More intense and wide screening of environmental matrices for the presence of *Toxoplasma* in different provinces of China is needed to enhance knowledge of *Toxoplasma* contamination of water resources and food matrices in this country.

## Methods

### Sampling and study area

**Figure 3:**
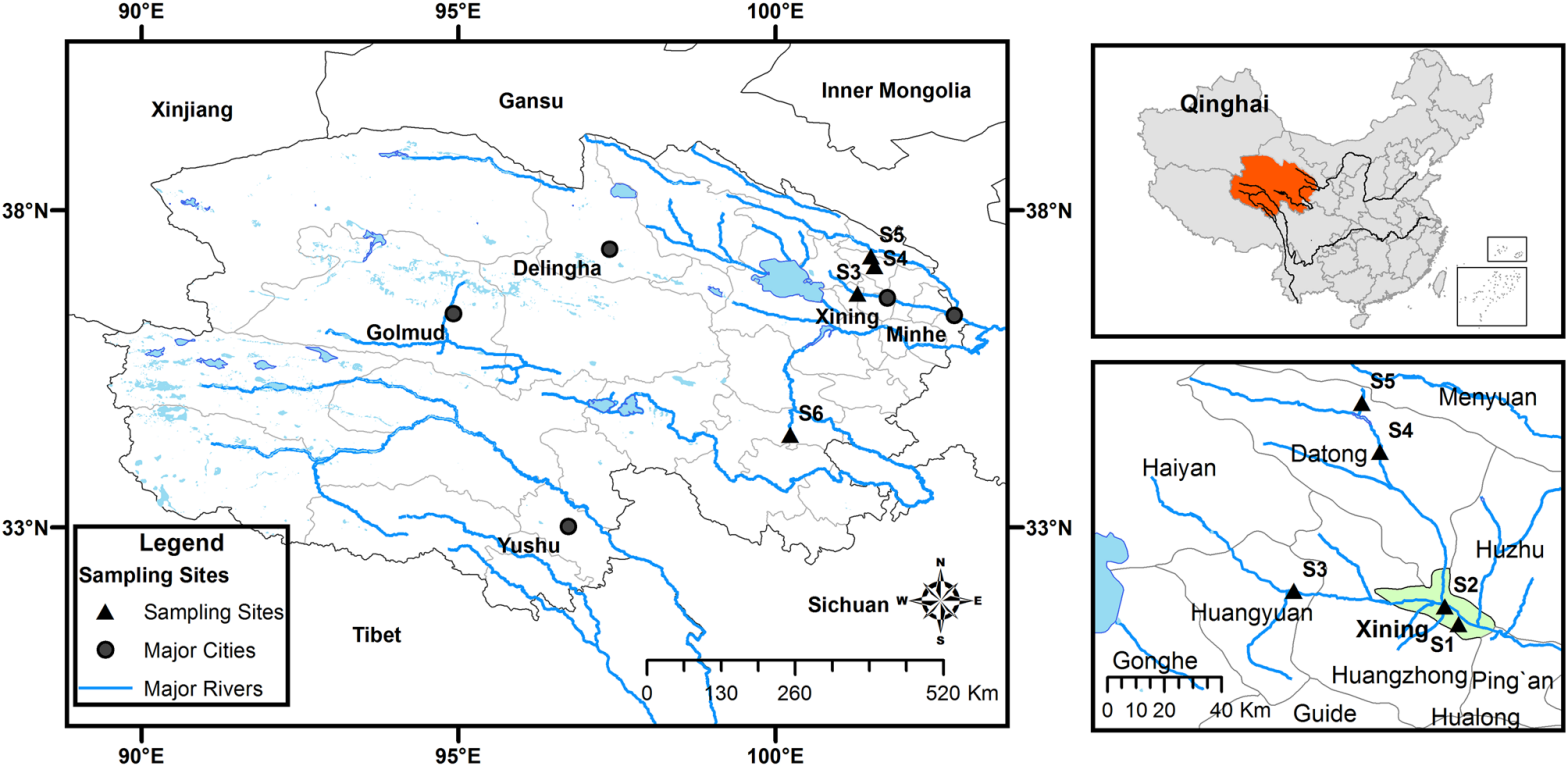
Sampling sites in Qinghai Province, P.R. China. S1-Baide slaughterhouse, S2-Xining WWTP, S3-Huangyuan WWTP, S4-Baoku River, S5-Chahan River, S6-Guoluo River (Modified by ArcGIS 10.2.2.3552 from CHGIS, Version 4” Cambridge: Harvard Yenching Institute, January 2007. Available at http://www.fas.harvard.edu/~chgis/; Contains modified Copernicus Service information [2015]).

A total of 214 water and wastewater samples were collected between January and October 2016 at various sites located in the Qinghai part of QTP, Western China (Fig. 3). Water and wastewater samples, 10 L in volume, were taken from two WWTPs, a slaughterhouse, and surface waters (Table 1). The samples originated from: Huangyuan WWTP (n = 101), Xining WWTP (n = 38), Baide slaughterhouse (n = 49), as well as Guoluo River (n = 10), Chahan River (n = 9), and Baoku River (n = 7) (Fig. 3, Table 1). The material was collected in sterile polypropylene vessels and transported to a laboratory in Xining, where it was immediately processed for analysis.

### Description of the sampling sites

Xining, Qinghai Province, P.R. China is the province capital with a population of 2.2 million people. It forms an extended administrative area including the counties of Datong, Huangyuan and Huangzhong and it is characterised by a densely populated metropolitan area of 1.2 million people, dominated by high-rise buildings, including rural and semi-rural districts with separate smaller cities. Xining WWTP is located in the city centre of Xining. It is one of the many secondary treatment facilities operating in the city, with a separate sewer system, processing domestic and industrial wastewater.

Huangyuan WWTP serves the nearby Huangyuan County. This is also a secondary treatment facility operating with a combined sewer system. It processes domestic wastewater, as well as storm water runoff. All of the wastewater samples from both WWTPs were collected before treatment, from WWTP influents.

The Baide slaughterhouse is a major facility, with 118 slaughtering production lines operating on a daily routine. It is situated in the outskirts of the Xining city centre. Water used for cleaning the processing area was sampled from the slaughterhouse floor, this water in a later processed by a municipal WWTP.

Chahan River, a tributary of Baoku, flows into Heiquan Reservoir across Baoku River, 80 km from Xining city centre. Baoku River is a major river which flows through Datong County into Huangshui He. Heiquan Reservoir is a possible source of drinking water in Qinghai Province. All the aforementioned WWTPs and rivers are connected with Huangshui He, a major Yellow River tributary flowing through the city of Xining. Finally, Guoluo River, the only site which is not connected with Huangshui He, is located in the pastoral area of Guoluo Tibetan Autonomous Prefecture, in the hinterland of QTP. This river serves directly as drinking water source, without treatment, for humans and animals, including domesticated animals (cattle, sheep, and dogs), as well as wild animals. Water samples were taken at the banks of rivers 1–2 m from the shore and from surface layer of water up to approximately 30 cm in depth.

### Isolation of *Toxoplasma gondii* from environmental material

In order to isolate and concentrate *T. gondii* from collected water and wastewater samples, two different protocols were applied depending on the type of investigated sample as given below.

A filtration method was employed to concentrate *T. gondii* oocysts from the environmental water samples^79^. Briefly, 10 L of surface water was filtered through membrane filters with a pore size of 1.2 µm using a 293-mm sanitary disc filter holder (Millipore) and CC-45 vacuum device (JAVAC, Australia). After filtration, the filters were washed at least three times (up to 5 times depending on the turbidity of water sample) with 15 mL 0.1% Tween 80 solution. The eluate and wash solutions were collected in sterile 50 mL conical tubes and centrifuged at 1,500 × *g* for 15 min. The supernatant was removed and the obtained pellet (1–2 mL, depending on water turbidity) was stored for further analysis.

For the wastewater samples collected from the WWTPs and slaughterhouse flocculation with Al_2_(SO_4_)_3_ according to^80^ was applied. Water pellets representing the end products of the processed samples were then used for DNA extraction.

### Microscopic investigation

Microscopic investigations of collected water samples (final pellet) were performed using fresh preparations observed under light microscope (Opta–Tech, Mn–358) using 40× magnification. Elements similar in size and structure to *T. gondii* oocysts^7,81,82^ were detected and recorded.

### Molecular detection of *Toxoplasma gondii*

#### DNA extraction

Prior to DNA extraction, the material (pellet) obtained from water samples was ten times frozen in liquid nitrogen and thawed at 85 °C in a water bath to improve the efficiency of DNA yield. Then, DNA extraction was performed using a commercial TIANamp Micro DNA Kit (DP 316) (Tiangen Biotech, Beijing, P.R. China) according to the manufacturer’s instructions. The extracted DNA was then stored at − 20 °C.

#### Specific detection of *Toxoplasma gondii* DNA

For the specific detection of *Toxoplasma gondii* DNA, real-time PCR was performed with the use of a pair of primers targeting a 129-bp fragment of the 35-fold repetitive B1 gene and the fluorescent-labelled TaqMan probe^83^. The amplification reaction mixture consisted of 12.5 μL of Real-Time 2× HS-PCR Master Mix Probe (A&A Biotechnology, Poland), 400 nM of each primer (Metabion, Germany), 80 nM of TaqMan probe (Genewiz SZ, Suzhou, P.R. China), and 5 μL of template DNA in a 25-μL reaction volume. Amplification was performed with an initial polymerase activation step (10 min at 95 °C), followed by 40 cycles of denaturation (15 s at 95 °C) and hybridisation/extension (1 min at 60 °C) in a Mx3005P thermocycler (Stratagene, USA). The obtained PCR products were analysed using MxPro QPCR Software. The cycle threshold (CT) value, determining the cycle number at which the reporter’s fluorescence exceeds the threshold value, was recorded. A sample was considered positive, if the CT value was <40. Next, to confirm the results of real-time PCR, both orientation cycle sequencing was performed using the primers Tox1 and Tox2 targeting fragment of B1 gene^84^ (Genewiz BJ, Beijing). The sequences obtained were analysed and compared with sequences from GenBank using GeneStudio Pro Software (GeneStudio, Suwanee, Georgia, USA). The results were analysed using GeneStudioTM Professional (GeneStudio, Inc., USA).

All PCR experiments were performed including the *T. gondii* positive control to ensure the correct functionality of the reaction, as well as negative controls to ensure that no PCR component had been contaminated. For the positive control in experiments, DNA isolated from tachyzoites of the parasite (the *T. gondii* RH strain), obtained from the National Institute of Hygiene, Poland has been used. Additionally, samples were retested for the presence of PCR inhibitors by mixing 4 μL of DNA template and 1 μL of internal positive control (IPC). Comparison of results of amplification obtained for samples containing a combination of IPC and template DNA with IPC alone allowed estimation of potential interference.

### Quantitative analysis, qPCR

Quantitative analysis was performed using the same primers and real-time PCR conditions as described above.

#### Standard curve

**Figure 4:**
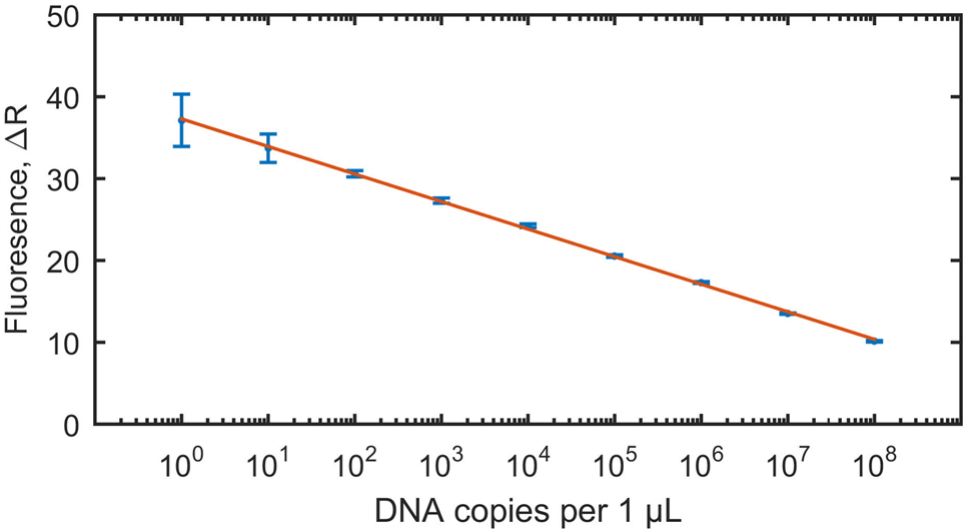
Standard curve generated from the amplification of nine dilutions of standard DNA in the range from one to 10^8^ DNA copies per one μL; each dilution was run in triplicates and standard deviation for each dilution was calculated and displayed. The standard curve served for the quantification of initial copy numbers of *T. gondii* B1 gene in investigated wastewater and water samples collected from the area of Qinghai Province, P.R. China. Figure was prepared using MATLAB R2015a.

The initial copy number of the detected *T. gondii* DNA in positive samples was calculated using qPCR based on a standard curve. First, to generate the standard template the insert gene (a fragment of the B1 gene amplified by PCR using the set of primers described above) was cloned into a plasmid. Next, three series of nine dilutions of standard DNA in the range from one to 10^8^ DNA copies per one μL were prepared and amplified. The standard curve was obtained by plotting the log of the initial template copy number against the CT value generated from each dilution (Fig. 4). Amplification of both the standard dilution series and the DNA isolated from positive samples was run on the same plate. Comparing the CT values of the unknown samples with the standard curve thus enabled the quantification of initial copy numbers^40^.

#### Determination of the number of *T. gondii* dispersive forms

The equivalent of *T. gondii* dispersive forms was estimated from the established initial number of copies of *T. gondii* DNA in each sample. Thus, the number of *T. gondii* cells (charge of the sample) was calculated using the following formula: CCS = N/35 × B, where N is the initial copy number determined using qPCR (per amount of DNA template taken for PCR); 35 is the number of copies of the B1 gene per one *T. gondii* cell; B is the multiplication factor referring to a total amount of DNA extracted from an investigated sample, in this study it is 10 because 5 µL of DNA template was taken for PCR from a total of 50 extracted per sample. Having CCS value, the number of *T. gondii* oocysts (oocysts charge of the sample) can be calculated as follows: OCS = CCS/A, where A=8 is the number of *T. gondii* sporozoites per one oocyst (the formula assumes that one sporulated oocyst contains eight fully developed sporozoites). Calculations were performed assuming that it is possible to detect a single *T. gondii* oocyst in a water suspension with real-time PCR, which has been validated in previous experiments^85^.

#### Genotyping

*T. gondii* genotypes were determined using multilocus PCR–RFLP assay with selected genetic markers: SAG1, SAG2, SAG3, BTUB, GRA6, c22-8, c29-2, L358, PK1 and Apico^74,75,86^. The set of reaction included nested PCR and restriction analysis of amplified products.

#### Amplification of genetic markers using nested PCR

The first step of nested PCR was performed using a set of external primers (Table 3) in a 25-μL reaction volume and the amplification reaction mixture consisted of 12.5 μL of the standard and ready-to-use PCR mixture 2xPCR Mix Plus High GC (A&A Biotechnology, Poland), 200 nM of each forward and reverse primer (Genewiz SZ, Suzhou, P.R. China), and 2 μL of template DNA. Amplifications were performed with an initial polymerase activation step (5 min at 95 °C), followed by 35 cycles of denaturation (30 s at 94 °C), primers annealing (1 min at 55 °C), strand extension (2 min s at 72 °C), and final extension (7 min at 72 °C). The second step of nested PCR reactions were performed using an internal set of primers (Table 1) under the following amplification reaction mixture conditions, 12.5 μL of the PCR mixture 2xPCR Mix Plus High GC (A&A Biotechnology, Poland), 400 nM of each primer (Genewiz SZ, Suzhou, P.R. China), and 2 μL of template DNA in a 25-μL reaction volume. Amplifications were performed according to the same protocol as in the first reaction, with the exception that annealing temperature was 60 °C.

**Table 3:** Overview of primers, enzymes and conditions used for multiplex multilocus nested PCR-RFLP typing of the *Toxoplasma gondii* isolates in the present study.

Marker		PCR primers (5ʹ–3ʹ)	Size (bp)	Restriction enzymes	Enzyme digestion conditions
SAG1^87^	SAG1 Ex F:	GTTCTAACCACGCACCCTGAG	390	Cfr13I + BfoI, Thermo Scientific (double digest)	37 °C 1 h
	SAG1 Ex R:	AAGAGTGGGAGGCTCTGTGA			
	SAG1 In F:	CAATGTGCACCTGTAGGAAGC			
	SAG1 In R:	GTGGTTCTCCGTCGGTGTGAG			
SAG2^75^	SAG2 Ex F:	GGAACGCGAACAATGAGTTT	546	HinfI + TaqI, Thermo scientific	37 °C 1 h + 65 °C 1 h
	SAG2 Ex R:	GCACTGTTGTCCAGGGTTTT			
	SAG2 In F:	ACCCATCTGCGAAGAAAACG			
	SAG2 In R:	ATTTCGACCAGCGGGAGCAC			
SAG3^87^	SAG3 Ex F:	CAACTCTCACCATTCCACCC	311	BcnI, Thermo Scientific	37 °C 1 h
	SAG3 Ex R:	GCGCGTTGTTAGACAAGACA			
	SAG3 In F:	TCTTGTCGGGTGTTCACTCA			
	SAG3 In R:	CACAAGGAGACCGAGAAGGA			
c 22-8^75^	C22-8 Ex F:	TGATGCATCCATGCGTTTAT	521	Alw26I + MboII, Thermo Scientific (double digest)	37 °C 1 h + 55 °C 1 h
	C22-8 Ex R:	CCTCCACTTCTTCGGTCTCA			
	C22-8 In F:	TCTCTCTACGTGGACGCC			
	C22-8 In R:	AGGTGCTTGGATATTCGC			
c 22-9^75^	C29-2 Ex F:	ACCCACTGAGCGAAAAGAAA	446	TaiI + RsaI, Thermo Scientific (double digest)	37 °C 1 h
	C29-2 Ex R:	AGGGTCTCTTGCGCATACAT			
	C29-2 In F:	AGTTCTGCAGAGTGTCGC			
	C29-2 In R:	TGTCTAGGAAAGAGGCGC			
L358^75^	L358 Ex F:	TCTCTCGACTTCGCCTCTTC	418	BsuRI + Hin1II, Thermo Scientific (double digest)	37 °C 1 h
	L358 Ex R:	GCAATTTCCTCGAAGACAGG			
	L358 In F:	AGGAGGCGTAGCGCAAGT			
	L358 In R:	CCCTCTGGCTGCAGTGCT			
BTUB^75^	BTUB Ex F:	TCCAAAATGAGAGAAATCGT	411	Bsh1285I + TaqI, Thermo Scientific (double digest)	65 °C 1 h
	BTUB Ex R:	AAATTGAAATGACGGAAGAA			
	BTUB In F:	GAGGTCATCTCGGACGAACA			
	BTUB In R:	TTGTAGGAACACCCGGACGC			
PK1^75^	L358 Ex F:	TCTCTCGACTTCGCCTCTTC	903	Eco88I + RsaI, Thermo Scientific (double digest)	37 °C 1 h
	L358 Ex R:	GCAATTTCCTCGAAGACAGG			
	L358 In F:	AGGAGGCGTAGCGCAAGT			
	L358 In R:	CCCTCTGGCTGCAGTGCT			
GRA6^75^	GRA6 Ex F:	ATTTGTGTTTCCGAGCAGGT	344	Tru1I, Thermo Scientific	37 °C 1 h
	GRAG Ex R:	GCACCTTCGCTTGTGGTT			
	GRA6 In F:	TTTCCGAGCAGGTGACCT			
	GRA 6 In R:	TCGCCGAAGAGTTGACATAG			
Apico^75^	APICO Ex F:	TGGTTTTAACCCTAGATTGTGG	640	AflII + DdeI, Thermo Scientific (double digest)	37 °C 1 h
	APICO Ex R:	AAACGGAATTAATGAGATTTGAA			
	APICO In F:	GCAAATTCTTGAATTCTCAGTT			
	APICO In R:	GGGATTCGAACCCTTGATA			

#### Restriction analysis of nested PCR products

In order to perform restriction analysis, 10 µL of PCR product was mixed with 2 µL of 10× digestive buffer and 1 U of restriction enzyme (each of the two restriction enzymes in case of double digestion) (Thermo Scientific, USA). The reaction was carried out according to the manufacturer’s instruction. RFLP products were analysed using a WD-9413B gel imaging analysis system (Beijing Liuyi Biotechnology, P.R. China) following electrophoresis on a 3% gel agarose (Biowest Regular Agarose G-10, Gene Company) stained with ExRed nucleic acid electrophoresis dye (Beijing Zoman Biotechnology, P.R. China).

### Statistical analysis

Statistical analysis of results including the confidence intervals for obtained percentages was performed using the Wilson Score without continuity correction. Calculations were performed using the Wilson Score Interval Calculator by Wolfram Alpha.

## Supplementary Information

Supplementary Figures.

## Data Availability

All data generated or analysed during this study are included in this published article.
