# Supplementary material for: Investigation of Toxoplasma gondii in wastewater and surface water in the Qinghai-Tibet Plateau, China using real-time PCR and multilocus genotyping
Source: Sci Rep. 2022 Mar 31;12:5428. doi: 10.1038/s41598-022-09166-0 (PMC8971506; doi:10.1038/s41598-022-09166-0)

Supplementary material

Legend to supplementary figures

Supplementary figure S1. *Toxoplasma gondii* – like oocyst visible in fresh preparation of the water sample No 125 collected from the Huangyuan WWTP, Qinghai Province, P.R. China. The picture presents element similar in size and structure to a sporulated *T. gondii* oocyst with sporocysts inside. The picture was cropped to improve the clarity of the presentation.

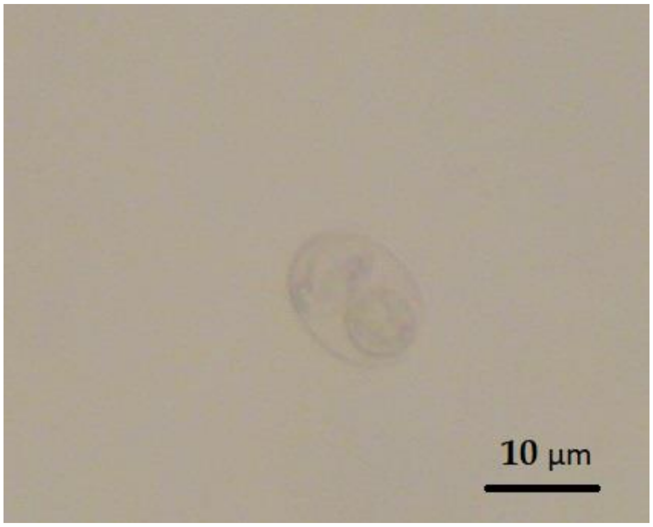

Supplementary Figures S2-5 represents raw gels (before cropping) obtained for genotyping of investigated water samples. Figures S1, S2, S3, and S4 corresponds respectively to the a, b, c, d, and e parts of the combined Figure 5 in the manuscript.

Supplementary Figure S2.

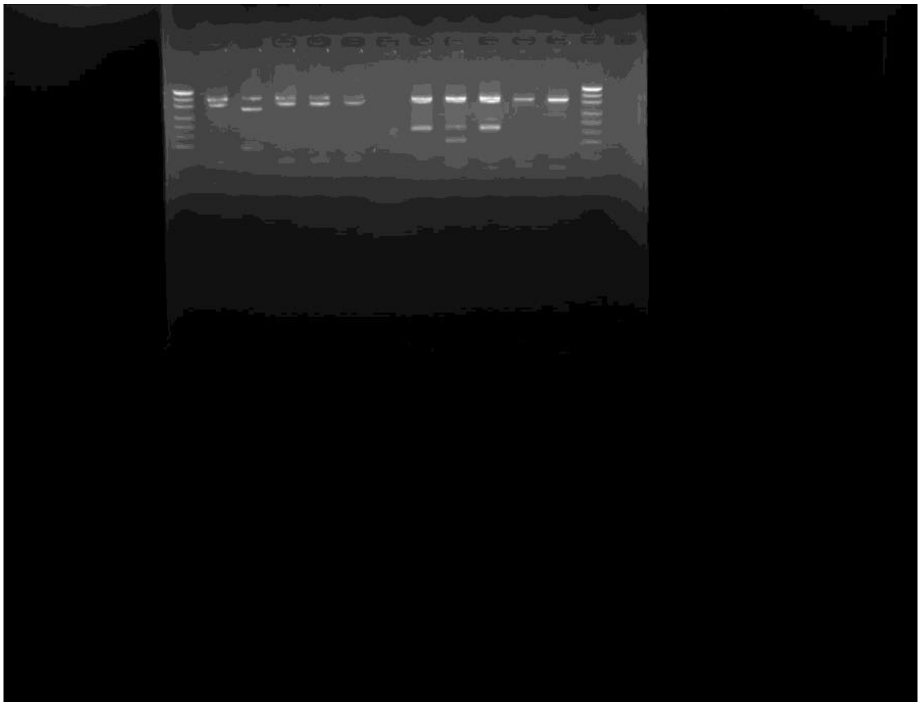

Supplementary Figure S3.

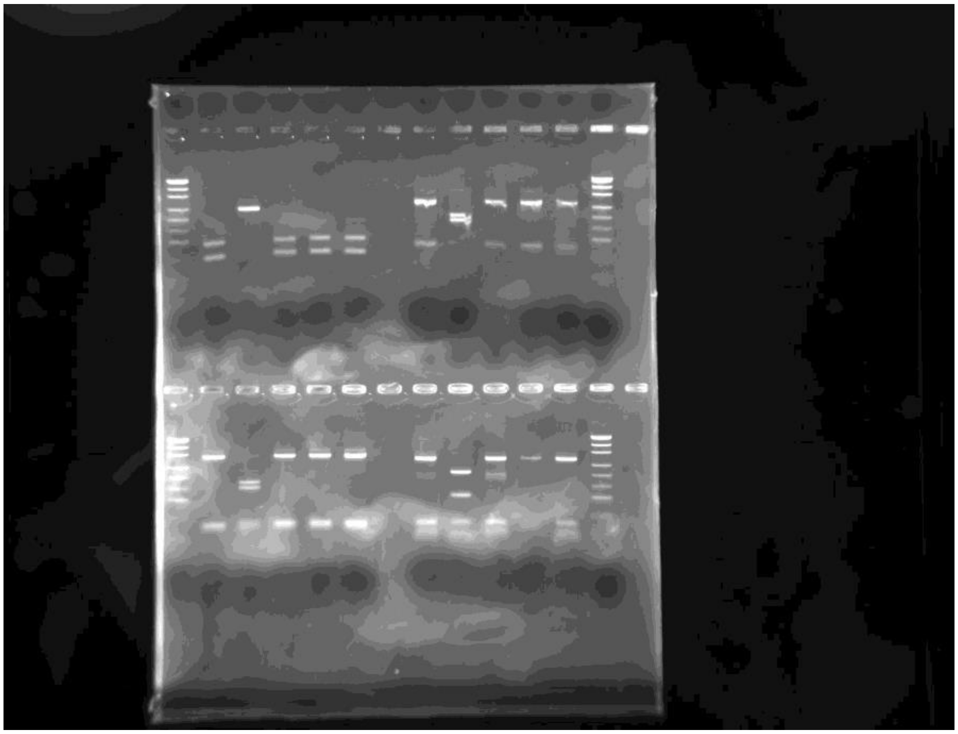

Supplementary Figure S4.

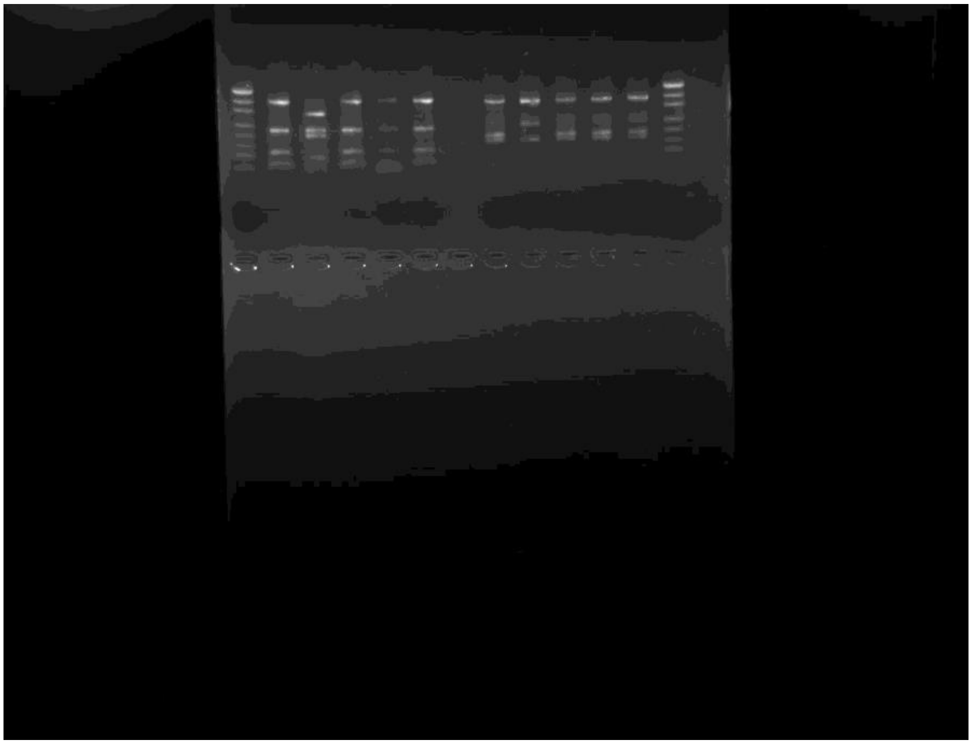

Supplementary Figure S5.

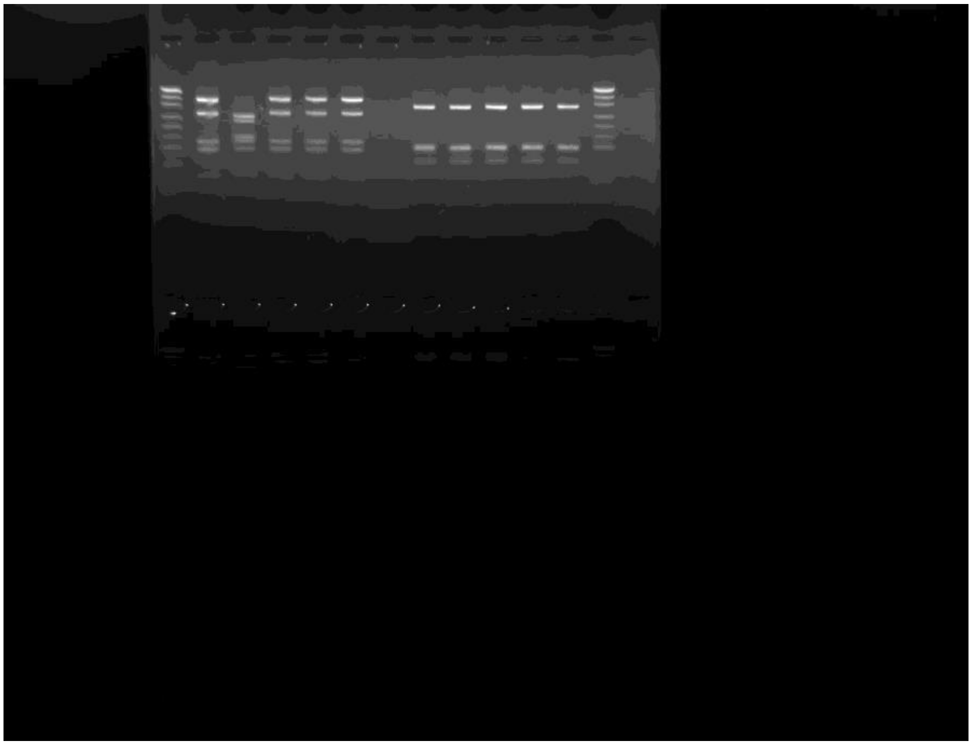

Supplement: Supplementary file 1 — Supplementary Figures. [file 41598_2022_9166_MOESM1_ESM.pdf]
